# Blumenthal technique and its modification: The glory of anterior chamber maintainer

**DOI:** 10.4103/0301-4738.58488

**Published:** 2010

**Authors:** Srikant Kumar Sahu, Sujata Das, Suryasnath Rath

**Affiliations:** Cornea and Anterior Segment Service, Hyderabad Eye Research Foundation, L V Prasad Eye Institute, Bhubaneswar - 751 024, Orissa, India; 1 Ophthalmic Plastic Surgery, Orbit and Ocular Oncology Service, Hyderabad Eye Research Foundation, L V Prasad Eye Institute, Bhubaneswar - 751 024, Orissa, India

Dear Editor,

Reading all the aspects of small-incision cataract surgery in a single issue was indeed a pleasure. Malik *et al*.[1] have described the original Blumenthal technique very eloquently and have added their personal modification.

We would like to put forth the following observation.

1. Small-incision cataract surgery (SICS) is considered as a cost-saving procedure and is suitable for the developing world.[2] Viscoelastic devices may not be required in most of the cases thus reducing the cost of surgery further. Under the anterior chamber maintainer (ACM) all the intraocular steps of SICS can be done with anterior chamber remaining deep. They include: capsulorrhexis, hydrodissection, prolapse of nucleus to anterior chamber, nucleus delivery, cortical matter removal, posterior capsule polish (with aspiration cannula attached to the syringe without plunger),[3] and intraocular lens implantation.
2. Use of Sheet's glide[4] can be conveniently omitted as they are single-use devices, and add to the cost. The authors have suggested an iris repositor as an alternate to Sheet's glide. The hydrostatic force of fluid in the anterior chamber guides the nucleus to the wound by just pressing the wound with McPherson forceps with tongues open, thus avoiding use of an iris repositor. Additionally, during this procedure the iris repositor being a hard instrument increases the chances of breaking the posterior capsule when it lies behind the lens material as the anterior chamber also marginally shallows.
3. When the nucleus is engaged in the incision and not being delivered out, a Sinskey hook (usually a part of the cataract tray) can be used to wheel out the nucleus. In our experience this is a better alternative than a 23-g needle, as suggested by the authors, to wheel out the nucleus. The needle is a sharp instrument; it can damage the surrounding structure including the wound integrity, specifically in the hands of a learner and the sharp edge of the needle could cut the nuclear material rather than wheeling it out.

We do agree with the authors that the Blumenthal technique is innovative, highly effective, reproducible in all grades of cataract, involves minimal intraocular manipulation and can be performed in the physiological condition of a closed system. We also agree with Thomas[5] who suggested that ACM allows anyone to learn SICS almost without a learning curve.
